# Phase Equilibrium Relations in the Binary System Barium Oxide-Niobium Pentoxide

**DOI:** 10.6028/jres.065A.036

**Published:** 1961-08-01

**Authors:** R. S. Roth, J. L. Waring

## Abstract

A large portion of the phase equilibrium diagram for the binary system barium oxide-niobium pentoxide has been constructed from observations of fusion characteristics and X-ray diffraction data. In the system five binary compounds were observed with BaO: Nb_2_O_5_ ratios of 5:2, 1:1, 6:7, 3:5, and 1:3 and a 6:1 compound was postulated. The 1:1 compound was found to melt congruently at 1,455 °C and have only one stable polymorph, although a second metastable polymorph can also be prepared. The 5:2 compound melts congruently at 1,542 °C; the 6:7, 3:5, and 1:3 phases melt incongruently at 1,330, 1,290, and 1,315 °C, respectively. The phase relations of the 6:1 compound could not be determined due to the reaction between this phase and platinum metal. No 2:1 compound was observed in this system.

## 1. Introduction

A study of phase relationships in the binary system BaO-Nb_2_O_5_ has been conducted as part of a program of fundamental phase equilibria studies of ceramic materials. The compound BaO·Nb_2_O_5_ has been previously reported by several workers [1,2]^1^, and one report exists in the literature of a compound having the formula 2BaO·Nb_2_O_5_ [3]. However, no systematic attempt to study the phase equilibrium relations in the entire binary system has been previously published.

X-ray diffraction data, together with the determination of the melting points of the compounds and of the solidus and liquidus temperatures at various compositions across the system have supplied data from which an equilibrium diagram has been constructed.

Owing to the reaction of BaO with Pt metal the high BaO portion of the system could not be studied by the present methods. This portion of the phase diagram, therefore, is necessarily left unknown, as is the case with all previously published phase diagrams involving BaO.

## 2. Sample Preparations and Test Methods

The following starting materials were employed for the preparation of specimens:

Nb_2_O_5_—high purity grade niobium pentoxide, manufacturer’s designation over 99.7 percent. Spectrographic analysis indicated less than about: 0.1 percent Si, 0.01 percent Fe, Sn, and Ti, 0.001 percent Ca and Mg. Cu was not determined because of Nb interference.
BaCO_3_—Reagent grade barium carbonate, manufacturer’s designation 99.3 percent purity.

For the preparation of batches the weight percent calculations were computed to within ±0.01 percent, with no corrections made for percentage purity of the raw materials except for loss on ignition. The starting materials were weighed to the nearest ±0.1 mg, in sufficient quantities to yield 3 g batches. Each batch was mixed in a mechanical shaker, and the bulk specimens were then ground in a mechanical agate mortar for 1 hr. At the termination of grinding a few drops of distilled water were added to facilitate pressing. The ground material was pressed into a disk in a ⅝ in. diameter mold at 10^4^ lb/in.^2^ The disks were placed on platinum foil and then calcined in air between 700 and 900 °C using an electrically heated furnace. No analyses were made after firing.

Following this preliminary heat treatment the disks were ground, reformed in a ⅝ in. mold at 1.5 × 10^4^ lb/in.^2^, and reheated to 1,100 °C for 8 hr.

Subsolidus as well as melting point data were obtained by the quenching technique on samples sealed in platinum tubes. An electrically heated, vertical tube furnace wound with 80 percent Pt-20 percent Rh wire was used. Temperatures were measured with a Pt versus Pt 10 percent Rh thermocouple which had been calibrated against the melting points of gold (1,063 °C) and barium disilicate (1,420 °C). The thermocouple was recalibrated several times during the course of the work. The first sign of adherence of the specimen to the platinum tube was interpreted as the first experimental evidence for the solidus temperature. This technique seemed justified for this particular system because of the marked fluidity of the liquid. The formation of a concave meniscus indicated the liquidus temperature.

Equilibrium was considered to have been obtained when the X-ray patterns of successively heated specimens showed no change. X-ray powder patterns were made using a high angle recording Geiger counter diffractometer and nickel-filtered copper radiation, with the Geiger counter traversing the specimen at l/4°/min and the radiation being recorded on the chart at 1° 2*θ*/in.

## 3. Compounds in the BaO-Nb_2_O_5_ System

### 3.1. Nb_2_O_5_

The stability relations of the various reported polymorphs of Nb_2_O_5_ have been summarized by several workers [4, 5, 6, 7]. Here it is only necessary to note that the so-called high temperature form of Nb_2_O_5_ was the only modification encountered in the present work. The indexed X-ray diffraction powder pattern and the unit cell dimensions of this monoclinic phase were listed in a previous publication on the PbO-Nb_2_O_5_ binary system [8].

### 3.2. Compounds 3BaO·5Nb_2_O_5_ and BaO·3Nb_2_O_5_

These two compositions have been observed to be essentially the end members of an “interrupted” solid solution series (a series involving a morphotropic phase transformation) having a distorted tungsten bronze type structure. The structure of the tungsten bronze type compounds will be discussed in section 5. It is only an academic question as to whether these compositions are true compounds or merely end members of the solid solution series. They were always observed to be single phase below the solidus and the compositions 5:8, on the high BaO side of 3:5, and 24:76, on the high Nb_2_O_5_ side of 1:3, show two phases. For these reasons the two end members are referred to as compounds for the sake of convenience. The X-ray patterns of the two compounds are very similar (tables 1 and 2), but have slightly different “superstructure” peaks indicating slightly different symmetries.

The diffraction peaks cannot be completely indexed on either the tetragonal or the orthorhombic modifications of the tungsten bronze structure observed for PbO·Nb_2_O_5_ [9]. The 3BaO·5Nb_2_O_5_ solid solution (including BaO:2Nb_2_O_5_) appears to be isostructural with PbO·2Nb_2_O_5_ [8]. The change in the position of the diffraction peaks in this solid solution region corresponds to an increase in the *c*/*a* ratio of the pseudotetragonal cell with decreasing BaO content, caused mostly by a decrease in *a* (with only a slight, if any, increase in *c*). The 1:3 solid solution on the other hand has a decrease in *c*/*a* ratio with decreasing BaO content, caused mostly by a decrease in c.

### 3.3. Compound 6BaO·7Nb_2_O_5_

A new compound has been observed at approximately this composition in the present study. This compound is very difficult to form as a single phase even at temperatures very near the solidus and may possibly be only metastable. However, the compound BaO·Nb_2_O_5_ is never observed in specimens heated below the solidus for compositions richer in Nb_2_O_5_ than the 6:7 mole ratio; therefore this new compound is concluded to be a true equilibrium phase. Its X-ray diffraction powder pattern is listed in table 3.

### 3.4. Compound BaO·Nb_2_O_5_

A compound of this composition was apparently first recorded by Krylov and Alekseev [10] as cubic with *a*=9.02 A; however, the X-ray diffraction pattern which they listed for this phase does not correspond to cubic symmetry. This reported phase had been formed by decomposition of a hydrate and was never encountered in the present work.

#### a. Metastable Hexagonal Modification

Francombe [11] first reported a hexagonal modification of BaO·Nb_2_O_5_ as “stable over a narrow temperature range below the melting point”. In the present work this hexagonal modification was not found at temperatures near the melting point. However, the hexagonal phase was encountered only under nonequilibrium conditions in the 1:1 BaO–Nb_2_O_5_ composition which was held for long periods of time at the relatively low temperatures of 900° to 1,100 °C. This modification is apparently not stable throughout the whole temperature range. The hexagonal phase is apparently a metastable intermediary structure between the orthorhombic BaO·Nb_2_O_5_ and the tungsten-bronze type structures. The X-ray diffraction powder pattern is given in table 4, with indices based on unit cell dimensions *a*= 12.07 A, *c*=3.95 A.

#### b. Stable Orthorhombic Modification

Apparently the only truly stable form of BaO·Nb_2_O_5_ is the orthorhombic modification. This phase was reported previously by others and a comparison of unit cell dimensions is given below.

**Table:** 

	*a*	*b*	*c*
			
	*A*	*A*	*A*
1957 Goodman [1]	12.24	10.29	7.90
1958 Coates and Kay [2]	12.16	10.27	7.78
1959 Francombe [12]	12.17	10.25	7.88
1960 Roth and Waring	12.194	10.268	7.856

The indexed X-ray diffraction powder pattern for orthorhombic BaO·Nb_2_O_5_ is listed in table 5.

### 3.5. Compound 5BaO·2Nb_2_O_5_

The compound 5BaO·2Nb_2_O_5_ is previously unreported. The X-ray diffraction powder pattern (table 6) can be completely indexed on the basis of a hexagonal unit cell with the dimensions *a*=5.794 A, *c*=11.784 A. The powder pattern was indexed on the basis of its similarity to the metastable hexagonal modification of BaO·Nb_2_O_5_ and the low temperature stable rhombohedral form of PbO·Nb_2_O_5_. However, this compound has many diffraction lines not allowed by the rhombohedral cell found in PbO·Nb_2_O_5_, and there are no lines in the powder pattern indicating the true cell has the larger $\left(a\sqrt{3}\right)$ dimension of the hexagonal counterpart of this rhombohedral cell.

### 3.6. Compound 6BaO·Nb_2_O_5_

A compound of this formula type has been previously reported by Brixner [13] for the BaO-Ta_2_O_5_ and SrO-Ta_2_O_5_ systems as having a cryolite type structure. The specimens were prepared by Brixner by heating in sealed evacuated silica tubes. The compound 6BaO·Nb_2_O_5_ was also observed by Francombe [12]. This phase has not been observed in the present study due to the reaction of BaO with Pt which occurs in compositions higher in BaO than the 5:2 ratio. However, certain compositions in the BaO-Nb_2_O_5_-Gd_2_O_3_ system [14] have been observed to show a single phase cubic solid solution of the cryolite type which is apparently based on the 6:1 compound and confirms its existence as a true binary phase. The high BaO portion of the binary system can probably only be studied in vacuum or inert atmosphere; however, the composition of the specimen holder for high temperatures is still a problem.

## 4. Discussion of Phase Equilibria

**Figure 1:**
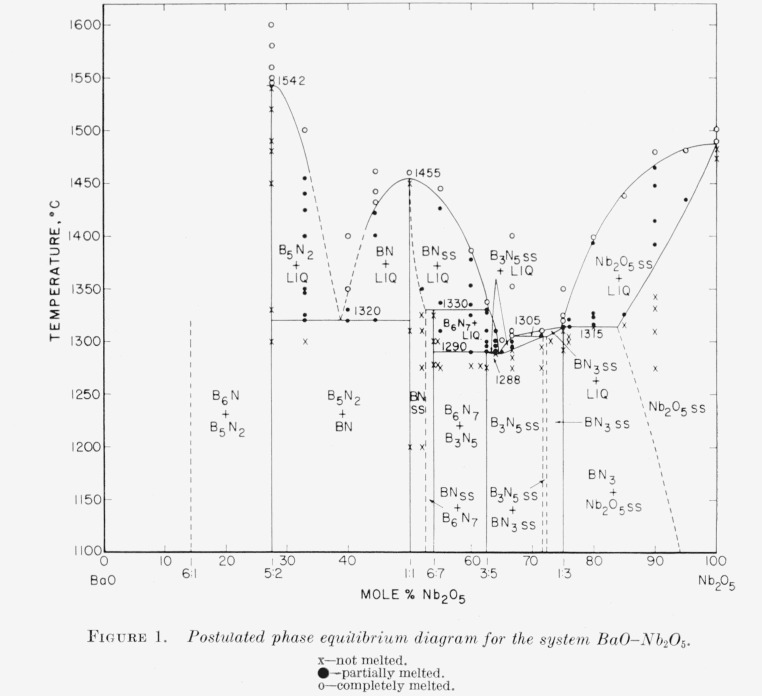
Postulated phase equilibrium diagram for the system BaO–Nb_2_O_5_. x—not melted. ●—partially melted. ○—completely melted.

The phase-equilibrium diagram of the binary system BaO-Nb_2_O_5_ is shown in figure 1. The data from which the diagram has been constructed are given in table 7. Most of the data was obtained from quenched specimens except where noted in the table. The designation Tet-ss in table 7 stands for the tetragonal tungsten bronze type structure, and signifies that the material which crystallized as “Tet-ss” must have been in the liquid state at the temperature from which the specimen was quenched (see discussion in section 5). The system contains two compounds which melt congruently, 5BaO·2Nb_2_O_5_ and BaO·Nb_2_O_5_; and three compounds which melt incongruently, 6BaO·7Nb_2_O_5_, 3BaO·5Nb_2_O_5_, and BaO·3Nb_2_O_5_. The melting point data for the compound 6BaO·Nb_2_O_5_ have not been determined.

The compound 5BaO·2Nb_2_O_5_ was found to melt congruently at about 1,542 °C. No phase transformations were observed in this composition. Compositions between 5BaO·2Nb_2_O_5_ and BaO·Nb_2_O_5_ when heated in air were found to contain only these two phases.^1^ A eutectic exists in the binary system between the two congruently melting compounds at 1,320 °C and is interpreted as occurring at about 62 mole percent BaO.

A compound of the ratio 2BaO·Nb_2_O_5_ was reported by Ismailzade [3] as tetragonal with *a*=10.928 A and *c*=11.167 A. No justification can be found in the present work for a compound at this ratio. From the unit cell dimensions listed, this phase would apparently be a tetragonally distorted pyrochlore structure. It can be deduced from the work of Isupov [15] that a pyrochlore type compound would not be expected to form in the BaO–Nb_2_O_5_ system.

The compound BaO·Nb_2_O_5_ was found to melt congruently at 1,455 °C. The hexagonal polymorph of this composition was not found above about 1,100 °C and was never found as a single phase (see table 7). The orthorhombic polymorph was present in nonequilibrium mixtures as low as 900 °C. As the hexagonal polymorph has never been shown to exist stably, it is concluded that this phase is merely metastable in the binary system.

A small amount of solid solution has been observed on the high niobia side of the BaO·Nb_2_O_5_ composition. This solid solution has not been entirely “quenched in” by the method employed and some nonequilibrium 3BaO·5Nb_2_O_5_ is always found in the X-ray patterns (see 48BaO:52Nb_2_O_5_, table 7). The solid solution apparently extends beyond 48:52 as the compound 6BaO·7Nb_2_O_5_ is never observed in long heat treatments of this composition. A two phase region of BaO·Nb_2_O_5_ solid solution and 6BaO·7Nb_2_O_5_ is postulated as extending from about 47.5 mole percent BaO to the 6:7 composition. As explained below, specimens in this composition range would always show three phases when quenched from below the solidus (BaO·Nb_2_O_5_+6BaO·7Nb_2_O_5_+3BaO·5Nb_2_O_5_).

The presence of the 3BaO·5Nb_2_O_5_ phase in the X-ray patterns of compositions between 1:1 and 6:7 has two possible explanations: (1) The BaO·Nb_2_O_5_ compound takes up excess Nb_2_O_5_ in soldid solution. This Nb_2_O_5_ cannot be retained on quenching and is exsolved. The 3BaO·5Nb_2_O_5_ forms instead of the 6BaO·7Nb_2_O_5_ because the former crystallizes very readily and the latter only with great difficulty. (2) The 3BaO·5Nb_2_O_5_ phase is formed as a nonequilibrium phase on heating and has not been transformed to the equilibrium 6BaO·7Nb_2_O_5_ even in 64 hr. Both explanations depend on the great ease of formation of the 3:5 bronze-type structure and the great difficulty of formation of the 6BaO·7Nb_2_O_5_ phase.

The compound 6BaO·7Nb_2_O_5_ was interpreted as melting incongruently to BaO·Nb_2_O_5_ solid solution plus liquid at 1,330 °C. Compositions between 6BaO·7Nb_2_O_5_ and 3BaO·5Nb_2_O_5_ were found to contain only these two phases below the solidus. The solidus corresponds to the incongruent melting temperature of the 3:5 composition. A solid of 3:5 composition was observed to melt incongruently to 6BaO·7Nb_2_O_5_ plus liquid at 1,290 °C. When quenched from any temperature above 1,330 °C, compositions between 6:7 and 3:5 showed the BaO·Nb_2_O_5_ solid solution instead of the 6BaO·7Nb_2_O_5_ compound.

From 37.5 mole percent BaO to 25 mole percent BaO a single phase solid solution area was observed. However, the end members of this solid solution series have slightly different “superstructure” peaks in their X-ray patterns (tables 1 and 2). A two phase area is therefore required somewhere in the solid solution series, at least at lower temperatures. This two phase area has not been observed experimentally but must exist between 28.57 mole percent BaO and 27 mole percent BaO or at about 72 mole percent Nb_2_O_5_. The existence of this two phase area is further indicated by the suggestion of a break in slope of the liquidus values occurring at 1,305 °C. This temperature corresponds to the solidus value projected for the 72 mole percent Nb_2_O_5_ composition. If the slope of the liquidus on the solid solution side is of opposite sign at the two ends of a solid solution area, a minimum is required. Experimentally, the difference between the 1,290 °C solidus of the 3BaO: 5Nb_2_O_5_ composition and the required minimum in the solidus could not be definitely proved. However, the minimum in the liquidus definitely occurs between 35 and 36 mole percent BaO and is shown in figure 1 as about 1,288 °C.

The compound BaO·3Nb_2_O_5_ was also interpreted as melting incongruently at about 1,315 °C to Nb_2_O_5_ solid solution plus liquid. However, this composition is just slightly incongruent. The nature of the melting was inferred from the observation that compositions slightly higher in Nb_2_O_5_, which contain two phases, began melting at experimentally the same temperature as was found for the 1:3 composition.

Compositions containing 85 mole percent, or more, Nb_2_O_5_ were found to begin melting at increasingly higher temperatures, indicating solid solution of BaO in Nb_2_O_5_. This solid solution is shown in figure 1 as extending to about 16 mole percent BaO at the solidus temperature. The solid solution could not be “quenched-in” at room temperature and specimens in this compositional range indicate two phases with little or no parameter change in the Nb_2_O_5_ phase. As indicated in table 7, the melting point of Nb_2_O_5_ was found to be about 1,487 °C, in reasonably good agreement with the value of 1,491 °C found by Holtzberg et al. [16].

## 5. Bronze-Type Solid Solution

From the location of the minimum in the liquidus at 35 to 36 mole percent BaO to the 1:3 composition (25 mole percent BaO), the liquid, when quenched, crystallizes as a metastable tetragonal bronze-type phase (table 7). This phase apparently also occurs in compositions on either side of this solid solution range, together with the other equilibrium phase, when quenched from above the solidus. However, any specimens quenched from below the solidus always exhibit a superstructure in the X-ray pattern of the bronze-type phase. The unit cell dimensions of the pseudotetragonal cell of the solid solution series are essentially the same as the unit cell dimensions of the true, metastable, tetragonal cell of the same composition. Apparently, the specimens quenched from the liquid exhibit “frozen-in” disorder, which gives rise to a higher symmetry.

The structure of the bronze-type phase is based on the structure of tetragonal potassium tungsten bronze described by Magneli [17]. This structure type has the general chemical formula A_6_B_10_O_30_, but often exhibits a deficiency of the *A*-type ions. It can be described as being made up of “octahedra coupled together in a rather intricate way forming rings or polygons of three, four, or five octahedra” [17]. The crystallographical formula can be written as $[A_{2}A_{4}^{\prime }][A_{2}B_{8}^{\prime }]O_{30}$. Thus there are two different A positions in the unit cell. The first position has two A ions surrounded by four octahedra and the second position has four A ions surrounded by five octahedra. The rings of three octahedra leave a vacancy in the lattice.

The superstructures occurring in the present bronze solid solutions might very likely be related to an ordering of Ba^+2^ ions in the A position which contains two ions surrounded by four octahedra. Thus the change in superstructure would be expected to occur at or near a composition which contains two Ba^+2^ ions per unit cell. As the exact ionic distribution in the 3:5 to 1: 3 BaO-Nb_2_O_5_ bronze solid solutions is not yet known, the chemical formulas can be written in several different forms. However, without the use of single crystal data nothing can be said about the exact nature of the various orderdisorder phases.

## 6. Summary

The system BaO-Nb_2_O_5_ was studied by means of solid state reactions, fusion characteristics, and X-ray diffraction data. The existence of six compounds in the system was postulated. They are 6BaO·Nb_2_O_5_, 5BaO-2Nb_2_O5 which melts congruently at about 1,542 °C, BaO·Nb_2_O_5_ which melts congruently at 1,455 °C, 6BaO-7Nb_2_O_5_ which melts incongruently at about 1,330 °C, 3BaO·5Nb_2_O_5_ which melts incongruently at about 1,290 °C, and BaO·3Nb_2_O_5_ which melts incongruently at about 1,315 °C. A eutectic occurs at about 62 mole percent BaO and 1,320 °C, and a minimum in a solid solution series occurs at about 35 to 36 mole percent BaO and 1,288 °C.

Complete solid solution was found between 3BaO·5Nb_2_O_5_ and BaO·3Nb_2_O_5_ although a change in superstructure symmetry occurred at about 28 mole percent BaO. A small amount of solid solution was noted on the high Nb_2_O_5_ side of BaO·Nb_2_O_5_, and Nb_2_O_5_ was found to accept about 16 mole percent BaO in solid solution at the solidus temperature. Neither of the partial solid solutions was quenchable, resulting in two phases at room temperature.

## Figures and Tables

**Table 1:** X-ray diffraction powder data for the composition *BaO* : *3Nb*_2_*O*_5_ (*CuK*α radiation)

*d*	*I*/*I*_0_	1/*d*^2^_obs_	1/*d*^2^_calc_	*hkl*^a^	*d*	*I*/*I*_0_	1/*d*^2^_obs_	1/*d*^2^_calc_	*hkl*^a^
8.82	13	0.0129	0.0129	110	2.384	6	0.1760	……….	……….
6.25	10	.0256	.0257	020	2.318	7	.1862	0.1863	250
5.58	2	.0321	.0321	120	2.283	7	.1919	.1920	241
4.41	12	.0514	.0514	220	2.1386	28	.2186	.2185	350
3.966	95	.0636	.0635	001	2.1105	4	.2245	.2241	431
3.943	65	.0643	.0643	130	2.0804	5	.2310	.2313	060
3.767	6	.0705	……….	……….	1.9840	55	.2541	.2541	002
3.709	15	.0727	……….	……….	1.9721	19	.2571	.2570	260
3.654	9	.0749	……….	……….	1.8595	25	.2892	.2891	360
3.461	65	.0835	.0835	230	1.7644	66	.3213	.3212	170/550
3.350	16	.0891	.0892	021	1.7486	18	.3270	.3269	451
3.234	50	.0956	.0956	121	1.7211	10	.3376	.3376	232
3.151	6	.1007	……….	……….	1.7154	9	.3398	.3405	270
3.117	8	.1029	.1028	040	1.6834	21	.3530	.3526	361
3.024	100	.1093	.1092	140	1.6592	32	.3632	.3633	142
2.941	72	.1157	$\{\;\begin{array}{l} .1149\\ .1157 \end{array}$	221	1.6445	15	.3698	.3697	332
				330	1.6163	14	.3828	.3826	242
2.857	7	.1225	……….	……….	1.6119	26	.3849	.3848	171/551
2.835	6	.1244			1.5584	7	.4117	.4112	080
2.796	90	.1279	.1278	131	1.5469	9	.4180	.4176	470/180
2.791	72	.1284	.1285	240	1.5120	8	.4374	.4369	280
2.723	9	.1348	……….	……….	1.4809	6	.4560	.4554	561
2.681	7	.1391	……….	……….	1.4598	8	.4692	.4690	380
2.608	50	.1471	.1470	231	1.4553	9	.4721	.4725	352
2.453	18	.1663	.1663	041	1.4511	8	.4749	.4747	081
2.448	17	.1669	.1671	150					
2.405	12	.1729	.1727	141					

a These *hkl* values are given by analogy to the tetragonal “bronze” structure. This is only a pseudocell, as there are many peaks which cannot be indexed on this basis. The pseudotetragonal cell has unit cell parameters of *a*=12.48 A, *c*=3.97 A.

**Table 2:** X-ray diffraction power data for the composition *3BaO* : *5Nb*_2_*O*_5_ (*CuK*α radiation)

*d*	*I*/*I*_0_	1/*d*^2^_obs_	1/*d*^2^_calc_	*hkl*^a^	*d*	*I*/*I*_0_	1/*d*^2^_obs_	1/*d*^2^_calc_	*hkl*^a^
8.81	10	0.0129	0.0128	110	2.453	15	0.1662	$\{\;\begin{array}{l} 0.1661\\ \;.1666 \end{array}$	041
6.25	14	.0256	.0256	020					150
5.60	6	.0319	.0320	120	2.365	5	.1788	.1789	331
3.964	51	.0637	$\{\;\begin{array}{l} .0635\\ .0641 \end{array}$	001	2.317	5	.1862	.1858	250
				130	2.140	20	.2180	.2178	350
3.860	5	.0671	……….	……….	2.0814	6	.2308	$\{\;\begin{array}{l} .2301\\ .2306 \end{array}$	151
									060
3.778	4	.0701	……….	……….	2.0001	7	.2499	.2493	521
3.619	10	0764	.0764	111	1.9835	40	.2542	.2542	002
3.545	9	.0796	……….	……….	1.8624	16	.2883	.2883	360
3.463	35	.0834	.0833	230	1.8461	6	.2934	.2942	061
3.350	25	.0891	.0892	021					
3.233	80	.0957	.0956	121				$\{\;\begin{array}{l} .3198\\ .3203 \end{array}$	261
									170/550
3.091	5	.1047	……….	……….	1.7518	17	.3259	.3262	451
3.026	100	.1092	.1089	140	1.7196	7	.3382	$\{\;\begin{array}{l} .3375\\ .3396 \end{array}$	232
2.945	52	.1153	$\{\;\begin{array}{l} .1148\\ .1153 \end{array}$	221					270
				330	1.6860	13	.3518	.3518	361
2.872	6	.1212	……….	……….	1.6598	27	.3630	.3631	142
2.796	90	.1279	$\{\;\begin{array}{l} .1276\\ .1281 \end{array}$	131	1.6450	12	.3696	.3695	332
				240	1.6135	19	.3841	.3839	171/551
2.636	6	.1439	……….	……….					
2.601	35	.1471	.1468	231					

a These *hkl* values are given by analogy to the tetragonal “bronze” structure. This is only a pseudocell, as there are many peaks which cannot be indexed on this basis. The pseudotetragonal cell has unit cell parameters of *a*=12.49 A, *c*=3.97 A.

**Table 3:** X-ray diffraction powder data for the compound *6BaO*·*7Nb*_2_*O*_5_ (*CuK*α radiation)

*d*	*I*/*I*_0_	*d*	*I*/*I*_0_
3.973	18	1.8599	8
3.506	15	1.8500	8
3.466	17	1.8219	16
3.270	23	1.7787	15
3.231	17	1.7650	8
3.170	100	1.7527	16
3.124	17	1.7299	9
3.034	100	1.7217	37
2.995	60	1.6992	10
2.947	12	1.6636	18
2.880	13	1.6568	25
2.797	25	1.6166	12
2.779	12	1.6044	11
2.686	28	1.5798	10
2.627	8	1.5511	5
2.605	26	1.5473	7
2.597	9	1.5290	5
2.392	5	1.5027	8
2.279	4	1.4988	8
2.2265	15	1.4830	8
2.1938	12	1.4545	8
2.1776	8	1.4020	6
2.1372	5	1.3542	7
2.0891	9	1.3150	13
1.9873	50	1.2854	6
1.9418	10	1.2370	9
1.9106	7	1.1809	9

**Table 4:** X-ray diffraction powder data for the metastable hexagonal form of *BaO*·*Nb*_2_*O*_5_ (*CuK*α radiation)

*d*^a^	*I*/*I*_0_	*hkl*^b^
5.22	12	200
3.95	38	001/210
3.15	100	201
3.02	100	220
2.904	10	310
2.791	8	211
2.613	7	301/400
2.393	11	221/320
2.180	75	401
2.043	5	321
1.974	50	002
1.881	15	510/112
1.846	9	501
1.769	50	212
1.765	40	421
1.745	45	600
1.716	14	430
1.697	13	511
1.653	33	222

a As this phase was found only in mixtures containing orthorhombic BaO·Nb_2_O_5_ the *d* values due to the later compound have been ignored for this table.

b The parameters upon which these *hkl* values are based are: *a*=12.07 A, *c*=3.95 A.

**Table 5:** X-ray diffraction powder data for the stable orthorhombic form of *BaO*·*Nb*_2_*O*_5_ (*CuK*_α_ radiation)

*d*	*I*/*I*_0_	1/*d*^2^_obs_	1/*d*^2^_calc_^a^	*hkl*
5.53	4	0.0327	0.0324	111
3.924	15	.0649	$\{\;\begin{array}{l} .0648\\ .0648 \end{array}$	002
				220
3.607	5	.0769	.0767	301
3.297	11	.0920	$\{\;\begin{array}{l} .0917\\ .0921 \end{array}$	202
				130
3.142	100	.1013	.1012	212
3.118	80	.1028	.1028	022
3.046	50	.1078	.1076	400
2.984	100	.1123	.1123	230
2.949	6	.1149	.1147	321
2.921	7	.1172	.1171	410
2.565	4	.1520	.1518	040
2.479	5	.1627	$\{\;\begin{array}{l} .1621\\ .1633 \end{array}$	331
				322
2.365	13	.1788	.1787	240
2.343	17	.1821	.1822	213
2.271	18	.1940	.1938	511
2.1801	29	.2104	.2104	422
2.1488	27	.2166	.2166	042
2.0924	10	.2284	.2285	341
2.0714	8	.2331	.2330	502
1.9689	18	.2580	.2578	432
1.9644	32	.2591	.2593	004
1.9262	9	.2695	.2697	531
1.8900	11	.2800	.2801	620
1.8398	12	.2954	.2956	214
1.8050	11	.3069	.3069	602
1.7775	12	.3164	.3164	612
1.7574	22	.3238	$\{\;\begin{array}{l} .3235\\ .3241 \end{array}$	513
				224
1.7446	47	.3286	.3289	252
1.7125	15	.3410	.3415	060
1.7025	7	.3450	.3449	622
1.6521	17	.3664	.3669	404
1.6415	32	.3711	.3715	234

a These values are based on the unit cell parameters: *a* = 12.194 A, *b* = 10.268 A, *c* = 7.856 A.

**Table 6:** X-ray diffraction powder data for the compound *5BaO*·*2Nb*_2_*O*_5_ (*CuK*α radiation)

*d*	*I*/*I*_0_	1/*d*^2^_obs_	1/*d*^2^_calc_^a^	Hexagonal *hkl*
4.61	5	0.0471	0.0469	101
3.827	9	.0683	.0685	102
3.093	100	.1045	.1045	103
2.897	100	.1192	.1192	110
2.540	8	.1550	.1549	104
2.453	7	.1663	.1661	201
2.358	12	.1799	.1800	005
2.308	25	.1877	.1877	202
2.1324	14	.2199	.2198	105
2.1143	100	.2237	.2237	203
1.9106	9	.2739	.2741	204
1.8298	49	.2987	.2990	106
1. 7080	62	.3428	.3429	213
1.6726	33	.3575	.3575	300
1.5963	7	.3924	.3926	107
1.5468	21	.4180	.4181	206
1.4487	27	.4765	.4767	220
1.3977	8	.5119	.5117	207
1.3644	22	.5372	$\{\;\begin{array}{l} .5373\\ .5375 \end{array}$	216
				305
1.3120	21	.5809	.5812	313
1.2583	3	.6316	.6316	314
1.1940	15	.7003	.7004	403
1.1933	14	.7022	.7025	119
1.1363	9	.7744	.7756	316
1.1050	10	.8189	.8195	323
1.0953	11	.8335	.8342	410
1.0925	9	.8378	.8393	1·1·10
1.0577	5	.8939	.8948	406
1.0311	5	.9405	.9408	309

a These values are based on the hexagonal unit cell parameters: *a*=5.794 A, *c*=11.784 A.

**Table 7:** Experimental data for compositions in the binary system *BaO*–*Nb*_2_*O*_5_

Composition^a^		Heat treatment^b^		Results	
		
BaO	Nb_2_O_5_	Temperature	Time	Physical observation	X-ray diffraction analyses^c^
					
*mole %*	*mole %*	*°C*	*hr*		
71.43	28.57	1300	16	No melting	5BaO·2Nb_2_O_5_.
(5:2)		1330	0.167	do	Do.
		1450	.5	do	Do.
		1480	.5	do	
		1490	17	do	
		1520	0.5	do	
		1540	.5	do	5BaO·2Nb_2_O_5_.
		1545	.333	Completely melted.	
		1550	.333	do	
		1560	.5	do	
		1580	.333	do	
		1600	.167	do	
		1650	.5	do	5BaO·2Nb_2_O_5_.
66.67	33.33	d1100	60	No melting	5BaO·2Nb_2_O_3_+Or−BaO·Nb_2_O_5_.
		1300	0.5	do	Do.
		1300	16	do	Do.
		1320	0.25	Just began to melt.	
		1325	.25	Some melting.	
		1346	.25	Partially melted.	
		1350	1.0	do	5BaO·2Nb_2_O_5_+Or−BaO·Nb_2_O_5_.
		1400	0.5	Considerably melted.	Do.
		1425	.25	do	Do.
		1440	.25	do	Do.
		1455	.25	do	Do.
		1500	.5	Completely melted.	Do.
60	40	1320	0.25	Just began to melt.	
		1330	.167	Considerably melted.	
		1350	.25	Completely melted.	
		1400	.25	do	
54.55	45.45	1320	0.167	Just began to melt.	
		1400	.25	Partially melted.	Or−BaO·Nb_2_O_5_+5BaO·2Nb_2_O_5_.
		1422	.25	do	
		1432	.25	Completely melted.	
		1442	.25	do	
		1461	.25	do	
50	50	d900	136	No melting	5BaO·2Nb_2_O_5_+Or−BaO·Nb_2_O_5_·+H−BaO·Nb_2_O_5_+3BaO·5Nb_2_O_5_.
		1050	64		Or−BaO·Nb_2_O_5_+H−BaO·Nb_2_O_5_·+5BaO·2Nb_2_O_5_+3BaO·5Nb_2_O_5_.
		1100	64	do	H−BaO·Nb_2_O_5_+Or−BaO·Nb_2_O_5_.
		d1200	1	do	Or−BaO·Nb_2_O_5_.
		d1310	1	do	Do.
		1450	0.33	do	Do.
		1460	0.33	Completely melted.	Do.
48	52	1100	64	No melting	H−BaO·Nb_2_O_5_+Or−BaO·Nb_2_O_5_+3BaO·5Nb_2_O5.
		1200	64	do	Or−BaO·Nb_2_O_5ss_+3BaO·5Nb_2_O_5_.
		1275	1	do	Or−BaO·Nb_2_O_5ss_+6BaO·7Nb_2_O_5_+3BaO·5Nb_2_O_5_.
		1310	16	do	Or−BaO·Nb_2_O_5ss_+3BaO·5Nb_2_O_5_ (trace).
		1325	0.33	do	Do.
		1350	.33	Just began to melt.	Do.
46.15	53. 85	1278	2	No melting	6BaO·7Nb_2_O_5_+Or−BaO·Nb_2_O_5ss_+3BaO·5Nb_2_O_5_.
(6:7)					
		1300	160.5	do	6BaO·7Nb_2_O_5_.
		1325	23	do	6BaO·7Nb_2_O_5_+Or−BaO·Nb_2_O_5ss_+3BaO.5Nb_2_O_5_.
45.45	54.55	1278	64	No melting	6BaO·7Nb_2_O_5_+3BaO·5Nb_2_O_5_.
		1300	0.833	do	Do.
		1300	160.5	do	Do.
45	55	1275	0.583	No melting	6BaO·7Nb_2_O_5_+3BaO·5Nb_2_O_5_.
		1310	1.0	Partially melted.	6BaO·7Nb_2_O_5_+Tet−ss.
		1337	0.167	do	Or−BaO·Nb_2_O_5_+Tet−ss.
		1426	0.167	Considerably melted.	
		1445	0.167	Completely melted.	
40	60	1277	15	No melting	3BaO·5Nb_2_O_5_+6BaO·7Nb_2_O_5_.
		1290	0.167	Just began to melt.	
		1292	1.0	do	Tet−ss+6BaO·7Nb_2_O_5_.
		1325	0.167	Considerably melted.	Do.
		1335	.167	do	Tet−ss+or−BaO·Nb_2_O_5_.
		1353	.167	do	Do.
		1378	.167	do	
		1386	.167	Completely melted.	
38.46	61.54	1277	15	No melting	3BaO·5Nb_2_O_5_+6BaO·7Nb_2_O_5_.
37.5	62.5	1275	0.583	No melting	3BaO·5Nb_2_O_5_.
(3:5)		1275	1.0	do	Do.
		1291	0.167	Just began to melt.	
		1296	.167	Considerable melting.	
		1300	.5	do	Tet−ss+6BaO·7Nb_2_O_5_.
		1310	.75	do	
		1327	1.0	do	
		1330	0.167	do	
		1337	.167	Completely melted.	Do.
36	64	1291	0.167	Just began to melt.	
		1296	.167	Considerably melted.	
		1301	1.0	do	Tet−ss+6BaO·7Nb_2_O_5_ (trace).
		1310	0.167	do	
35	65	1290	0.167	Just began to melt.	
		1301	.167	Completely melted.	
33.33	66.67	1277	.5	No melting	
		1285	.167	do	
		1292	.333	do	3BaO·5Nb_2_O_5_SS.
		1295	.033	Just began to melt.	
		1300	1.0	Partially melted.	3BaO·5Nb_2_O_5_SS (+Tet−ss?).
		1306	0.167	Completely melted.	
		1310	.083	do	
		1352	.25	do	
		1400	.167	do	Tet−ss.
28.57	71.43	1275	.583	No melting	3BaO·5Nb_2_O_5_ss.
		1295	.033	do	
		1306	.167	Partially melted.	
		1310	.5	Completely melted.	Tet−ss.
27	73	1301	16	No melting	BaO·3Nb_2_O_5_ss.
25	75	1292	1.0	No melting	
(1:3)		1310	0. 333	do	BaO·3Nb_2_O_5_.
		1311	.167	do	
		1314	.083	Partially melted.	BaO·3Nb_2_O_5_ (+Tet−ss?).
		1315	.167	do	
		1320		Completely melted.	
		1325	.25	do	Tet−ss.
		1350	.083	do	Do.
24	76	1300	.167	No melting	BaO·3Nb_2_O_5_+Nb_2_O_5_ (trace).
		1305	.167	do	
		1315	.167	Just began to melt.	
		1321	.167	Partially melted.	
20	80	1314	.167	No melting	
		1316	.167	Just began to melt.	
		1323	.167	Partially melted.	
		1327	.167	do	
		1394	.167	Considerably melted.	
		1399	.167	Completely melted.	
15	85	1316	.167	No melting	
		1326	.167	Just began to melt.	
		1438	.167	Completely melted.	
10	90	1275	.583	No melting	Nb_2_O_5_+BaO· 3Nb_2_O_5_.
		1310	.75	do	Do.
		1332	.25	do	
		1343	.25	do	
		1392	.333	Just began to melt.	
		1415	.167	Partially melted.	
		1448	.25	Considerably melted.	
		1465	.333	do	
		1480	.167	Completely melted.	
5	95	1439	.167	Just began to melt.	
		1482	.167	Completely melted.	
0	100	1474	.333	No melting	
		1483	.5	do	
		1490	.167	Completely melted.	
		1502	.167	do	

See footnotes at end of table.

a All specimens containing more than 71.43 mole percent BaO reacted with the Pt containers.

b Unless otherwise indicated all specimens were quenched in sealed Pt tubes.

c The phases identified are given in the order of the amount present at room temperature. The phases are not necessarily those present at the temperature to which the specimen was heated. H—hexagonal, Or—orthorhombic, Tet—tetragonal, ss—solid solution.

d These specimens were heated in air on Pt foil, and slow cooled.

## Footnotes

1 Figures in brackets indicate the literature references at the end of this paper.

1 When heated in a helium atmosphere, however, the composition 6BaO·5Nb_2_O_5_ contained essentially a single phase bronze type structure which would probably correspond to the ternary compound Ba_6_^+2^(Nb_8_^+5^Nb_2_^+4^)O_30_.
